# Neural Correlates of Odor Learning in the Presynaptic Microglomerular Circuitry in the Honeybee Mushroom Body Calyx

**DOI:** 10.1523/ENEURO.0128-18.2018

**Published:** 2018-06-18

**Authors:** Joachim Haenicke, Nobuhiro Yamagata, Hanna Zwaka, Martin Nawrot, Randolf Menzel

**Affiliations:** 1 Institute of Biology, Freie Universität Berlin, Berlin 14195, Germany; 2 Bernstein Center for Computational Neuroscience, Humboldt-Universität zu Berlin, Berlin 10115, Germany; 3 Graduate School of Life Sciences, Tohoku University, Sendai 980-8577, Japan; 4 Molecular and Cellular Biology, Harvard University, Cambridge, MA 02138; 5 Computational Systems Neuroscience, Institute of Zoology, Universität zu Köln, Köln 50931, Germany

**Keywords:** calcium imaging, honeybee, learning and memory, mushroom body, projection neurons

## Abstract

The mushroom body (MB) in insects is known as a major center for associative learning and memory, although exact locations for the correlating memory traces remain to be elucidated. Here, we asked whether presynaptic boutons of olfactory projection neurons (PNs) in the main input site of the MB undergo neuronal plasticity during classical odor-reward conditioning and correlate with the conditioned behavior. We simultaneously measured Ca^2+^ responses in the boutons and conditioned behavioral responses to learned odors in honeybees. We found that the absolute amount of the neural change for the rewarded but not for the unrewarded odor was correlated with the behavioral learning rate across individuals. The temporal profile of the induced changes matched with odor response dynamics of the MB-associated inhibitory neurons, suggestive of activity modulation of boutons by this neural class. We hypothesize the circuit-specific neural plasticity relates to the learned value of the stimulus and underlies the conditioned behavior of the bees.

## Significance Statement

In order to understand memory processing in the brain, it is important to identify the synaptic locations and activities where memory information is stored. This requires monitoring neuronal activity in behaving animals, a technically very demanding task especially in tiny insects. Here, we succeeded to measure neuronal activity from restrained yet behaving honeybees. We recorded the activity of olfactory projection neurons (PNs) from the mushroom body (MB), an insect brain center for learning and memory, while bees are performing an olfactory reward learning task. We found that the amount of neural plasticity correlates with the quantitative expression of a learned behavior to the sugar-rewarded odor. Our results contribute to the understanding of the physiologic basis of memory formation in an insect brain.

## Introduction

Mechanistic understanding of the neural processes of learning and memory requires the identification of the cellular compositions where learning-related plasticity takes place. In searching for such “memory traces”, the classical olfactory conditioning whereby an animal learns the association between an odor and a reward or punishment has been used widely (Wilson and Stevenson, 2003; Davis, 2004). Thanks to its robust behavioral performance and relatively simple brain circuitry, the honeybee has been studied intensively as a model organism for this type of olfactory learning (Menzel, 2001; Giurfa and Sandoz, 2012). To date, a rich body of evidence supports the interpretation that the sequential and parallel activity of the two olfactory centers, the antennal lobe (AL) and the mushroom body (MB), play a key role in the formation and recall of some forms of olfactory memory in the brain of bees (Hammer and Menzel, 1998; Menzel, 2012) and flies (Heisenberg, 2003; Yu et al., 2004; Scheunemann et al., 2012; Scholz-Kornehl and Schwärzel, 2016). The exact localization of learning-related plasticity in that circuitry, however, remains elusive.

In this study, we investigated the network activity of the terminal boutons of the olfactory projection neurons (PNs) in the MB calyx on learning. The MB calyx is a main input site and is formed by the dendritic arbors of the intrinsic Kenyon cells (KCs; Mobbs, 1982). Parallel axonal fibers of these KCs form lobe structures, along which each cell forms numbers of en passant output synapses. The calyx can be anatomically separated into three subdomains. One such domain is the lip region, the main olfactory input site, containing numerous synaptic inputs from the PN boutons to the dendritic arbors of the KCs (Ganeshina and Menzel, 2001). The lip is also innervated by the octopaminergic neuron that conveys the reinforcing properties of the rewarding unconditioned sugar stimulus during associative learning (Hammer, 1993). These circuit conditions make the lip region a unique site of odor and sugar reinforcement convergence. An additional information flow into the lip occurs through the GABAergic inhibitory feedback neurons in the protocerebrum-calyx tract (PCT) that originate in the MB lobes and project back into the calyx (Grünewald, 1999a). These neurons alongside with other MB output neurons (MBONs) exhibit learning-dependent plasticity (Mauelshagen, 1993; Grünewald, 1999b; Okada et al., 2007; Strube-Bloss et al., 2011; Filla and Menzel, 2015). In *Drosophila*, it is believed that memory information is mainly stored in the output synapses between the KCs and MBONs (Heisenberg, 2003), and different MBON channels undergo learning-dependent plasticity by their own rules (Aso et al., 2014; Hige et al., 2015). In honeybees, however, both the anatomic convergence of the CS (odor) and the US (sugar reward) pathways and the learning-specific plasticity of the KC spines (Szyszka et al., 2008) indicate associative olfactory plasticity also in the input site of the MB. Within the lip, these neural compositions locally form numbers of synaptic hubs, called microglomeruli (Ganeshina and Menzel, 2001). The PN boutons form the core of these microstructures by integrating the inhibitory input from recurrent PCT neurons (Grünewald, 1999a; Filla and Menzel, 2015), local excitatory feedback from the KCs (Ganeshina and Menzel, 2001), and the octopaminergic reward input (Hammer, 1993; Sinakevitch et al., 2013). This specific circuit configuration of the presynaptic PN boutons prompted us to think that they may play a key role in olfactory learning and are subject to experience-dependent plasticity.

Here, we studied neural plasticity of the PN boutons in the MB calyx with an emphasis on its relation to the learning rate on the level of the individual animal. The learning performance was monitored by the conditioned proboscis extension response (PER) while odor responses in individual PN boutons were measured by Ca^2+^ imaging. During learning, individual boutons showed an increase or decrease of their Ca^2+^ responses to both the rewarded (CS+) and the unrewarded stimulus (CS-). No significant differences between CS+ and CS- odors were observed in the way the responses were up- or down-regulated. However, the response pattern similarity decreased after learning. Moreover, the amount of neural plasticity in absolute terms (i.e., unsigned increase and decrease) induced for the CS+ is strongly and positively correlated with the learning rate across individual animals. The temporal profile of the induced changes matched the Ca^2+^ response dynamics of the inhibitory GABAergic feedback neurons, suggestive of associative activity modulation of boutons by these inhibitory neurons. We hypothesize that the observed plasticity in olfactory presynaptic terminals relates to the learned value of the stimulus, signifies short-term memory, and underlies the conditioned response (CR) behavior of the bees.

## Materials and Methods

### Preparation and dye loading

Bees were prepared as described previously (Yamagata et al., 2009). In short, foraging female worker bees were collected, chilled and fixed in recording chambers with wax. The head capsule was opened and a mixture of the solid Ca^2+^-sensitive dye fura-dextran (10,000 MW, Invitrogen) and the lysine fixable dye tetramethylrhodamine-dextran (10,000 MW, Invitrogen) was injected into the brain aiming for the soma cluster of the PNs of the lateral AL tract (l-ALT). Then the head capsule was closed, and the bees were fed until satiation and kept at 17–20°C for 8–24 h. Before measurements, the legs and wings were cut, and the abdomen, thorax, and mandibles were immobilized with wax. The antennae were fixed with n-eicosane and the calyces of the MB were exposed for measurements. Kwik-Sil adhesive (World Precision Instruments, Inc.) was poured into the head capsule to completely stabilize the brain. After sealing the gaps with Vaseline, the recording chambers were filled with Ringer solution (130 mM NaCl, 7 mM CaCl_2_, 6 mM KCl, 2 mM MgCl_2_, 160 mM sucrose, 25 mM glucose, and 10 mM HEPES, pH 6.7, 500 mosmol).

### Odor stimulation and conditioning

In most bees, 2-octanone and octanal (Sigma) diluted to 10^−2^ with paraffin were used. 1-Hexanol and 2-octanol (Sigma) were used either with or without dilution (10^−2^ and 5 × 10^−2^, respectively). Whole during experiments, the bees were exposed to a constant air stream. Injection of the odorant (40 μl soaked with 1 × 2 cm filter paper) into the constant air stream was switched on and off by a computer-controlled solenoid valve (Galizia et al., 1997; Peele et al., 2006; Haehnel and Menzel, 2010, 2012). Odors were presented for 3 s at an interstimulus interval and an intertrial interval of 90 s.

**Figure 1.**
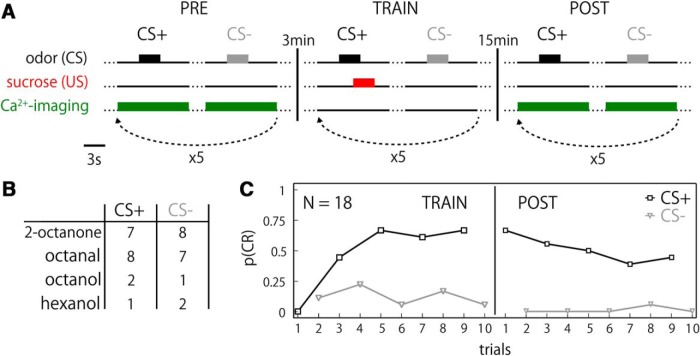
Experimental paradigm and behavioral results. ***A***, An experimental schedule. In the pre-training (PRE) and post-training (POST) phases, two odors were applied in pseudo-random order, at least five times each. The training phase (TRAIN) started with the sucrose-rewarded odor (CS+), alternating with the unrewarded odor (CS-). Ca^2+^ imaging was performed during PRE and POST phases. Intervals between odor stimuli and between training repetition cycles were both 90 s. ***B***, The numbers of animals trained with the specified odors for CS+ and CS-. Different odors were equally used as rewarded and unrewarded stimulus. ***C***, Behavioral data for all bees (*N* = 18) during TRAIN (trials 1–10) and POST (trials 11–20) phases. The probability of CRs by a population [p(CR)] rapidly increased and saturated after two pairings of odor and sugar. A small fraction of bees initially generalized toward CS-.

Before starting conditioning, the proboscis extension response (PER) to the sugar stimulus of each bee was checked and only responding animals were moved to the recording site and given some minutes to rest. The protocol for the classical conditioning experiment (Bitterman et al., 1983; Matsumoto et al., 2012) followed the design given in Figure 1*A*. In the pre-training phase (PRE), each bee was exposed to two different odors for five to eight times in a pseudo-random order. After an interval of 3 min, bees were conditioned to one of the odors (CS+) by forward pairing the odor with an unconditioned reward stimulus (US) consisting of a drop of 30% sucrose. Conditioning always started with the rewarded odor (CS+) and the unrewarded control odor (CS-) was alternately presented (5–10 trials). After a 15-min retention, bees were again exposed to both odors for at least five trials (five to eight trials) in a pseudo-random order (POST). In all three phases, we monitored the animals’ conditioned response (CR) as expressed in the PER by visual inspection. Only a complete extension of the proboscis was regarded as the CR. At the end of each experiment, the sugar response of each bee was tested again and only responding animals were included in the analyses.

### Ca^2+^ imaging

Ca^2+^ measurements were performed at room temperature with a sampling rate of 5 Hz, using a TILL-Photonics imaging setup mounted on a fluorescence microscope (Zeiss Axioskop). Fura was alternately excited at 340 and 380 nm. Exposure times were 15 and 60 ms, respectively. Each measurement started 3 s before stimulus onset and lasted for 10 s. Images were acquired through a 60×/0.9 NA water dipping objective (Olympus), a 410-nm dichroic mirror and a 440-nm-long pass filter with an Imago CCD camera (640 × 480 pixels, 4× binned on chip to 160 × 120). Pixel size was 1.47 × 1.47 μm, which allowed a resolution of single boutons of PNs. Approximately 1/40th of the calyx ring neuropil was imaged focusing on the frontal margin of the calyx. We performed imaging during the PRE and the POST phase while Ca^2+^ responses were not recorded during the training phase due to movement artifacts of the brain by sugar stimulation. For imaging during the POST phase, animals were selected according to their behavioral performance during training. Consequently, only a small fraction of non-learners was imaged.

### Confocal microscopy

After Ca^2+^ measurements, the brain was dissected and fixed in 4% formaldehyde in Millonig’s buffer overnight at 4°C. The brain was then rinsed in saline, dehydrated in ethanol, cleared in methyl salicylate, set into a chamber filled with methyl salicylate and observed with a confocal microscope (Leica TCS SP2; Leica). The excitation wavelength was 543 nm using a green HeNe laser. The entire brain was scanned with a 10×/0.4 NA air objective (Olympus). Where necessary, the AL was scanned with a 20×/0.70 NA air objective (Olympus) and the MB calyx was scanned with a 63×/1.32–0.6 NA oil objective (Olympus). Morphologic images were acquired as an averaged raw fluorescence image of 380 nm during the measurements and was later unsharp mask-filtered in Photoshop (Adobe).

### Identification and pre-processing of bouton activity

**Figure 2.**
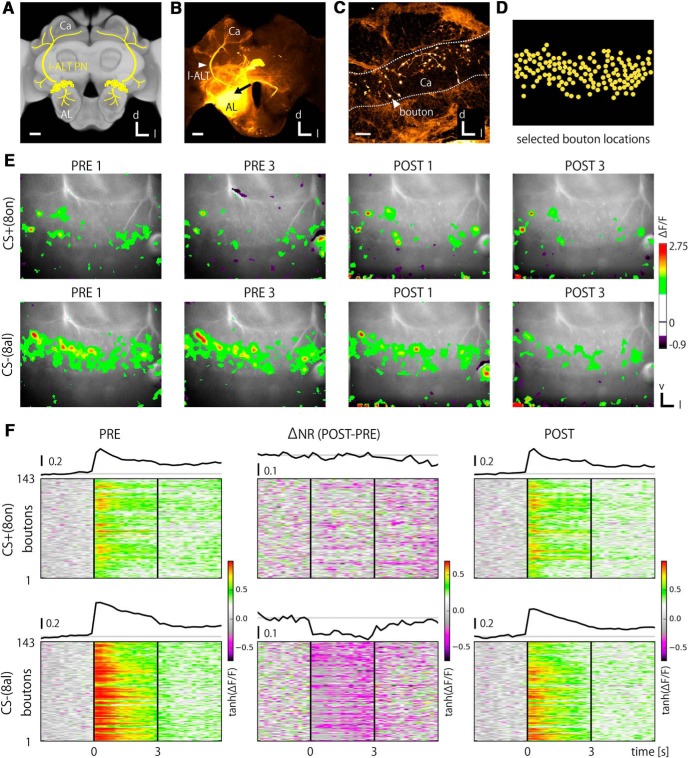
Bouton responses and their temporal properties. ***A***, Frontal view of the honeybee brain with schematic overlay: uniglomerular PNs (yellow) connect the AL with the MB calyx (Ca) via the l-ALT (arrowhead). d: dorsal, l: lateral. ***B***, A confocal image of stained brain viewed frontally. PN somata were stained with a mixture of the fura-dextran and the rhodamine dextran. Arrow, a site of injection. Scale bars, 100 μm. ***C***, A confocal image of rhodamine fluorescence in the MB calyx. Individual boutons are visualized. Scale bar, 20 μm. ***D***, An example of identified locations of synaptic boutons based on their Ca^2+^ responses (bee #11, also in Figs. 3, 4). ***E***, Color-coded Ca^2+^ responses superimposed on raw fluorescence images of PN boutons for two trials during PRE (left) and POST (right) phases in response to rewarded (upper row) and unrewarded (lower row) odors. The same animal as in ***D***. 8on: 2-octanone, 8al: octanal. ***F***, An example of temporal dynamics of Ca^2+^ activity for individual boutons (heat map) and their average traces (black curve) for CS+ (upper) and CS- (lower) odors in a representative bee #11. Changes between PRE (left) and POST (right) phases were also calculated per boutons (middle). Data were transformed by the hyperbolic tangent function (Extended Data Fig. 2-1; see also Materials and Methods). Individual bouton traces show the trial-averaged activities based on signal quality check (Extended Data Figs. 2-2, 2-3).

Recorded videos of Ca^2+^ responses were preprocessed in IDL (RSI) using custom scripts as described in Yamagata et al. (2009). A mean of 15 frames during a single odor stimulation was calculated and displayed as a false-color image (Fig. 2). A spatial low-pass filter (5 × 5 pixels) was applied to these images for better visualization. Individual boutons were determined as isolated activity spots (21 pixels) in the false-color images. For each bouton a response trace was calculated by averaging the signal of an activity patch without any filtering and correction.

10.1523/ENEURO.0128-18.2018.f2-1 Extended Data Figure 2-1 Transformation of Ca^2+^ responses. ***A***, Tangens hyperbolicus transformation of ΔF/F signal. Black curve represents transformation across the total range of ΔF/F values encountered in all boutons of bee #9. ***B***, Transformation of exemplified single bouton activities. Black shades indicate odor stimulation for 3 s. Large excitatory response values are compressed by the transformation while smaller excitatory and inhibitory response values around zero are enhanced. ***C***, Color code visualization of all 107-bouton response in bee #9 in response to the CS+ odor before (PRE) and after (POST) training and the difference (POST-PRE). The top row shows the untransformed ΔF/F response signals. The color map spans the complete data range across all boutons as shown in ***A***, top. The bottom row shows the transformed tanh (ΔF/F) response signals. The color map spans the complete range of transformed data across all boutons as shown in ***A***, right. This leads to a compression of strong excitatory responses (reddish colors) and increases the dynamic color range for weaker excitatory and inhibitory responses. In effect, we observe clear excitatory and inhibitory responses across many boutons. The same classical “jet” 64-bit color map (as shown in ***A***) is used for upper and lower row. Download Figure 2-1, TIF file.

10.1523/ENEURO.0128-18.2018.f2-2 Extended Data Figure 2-2 Response statistics within and across experimental phases. ***A***, Distributions of pairwise Euclidean distances of odor responses of different trials within and between different experimental phases. More similar bouton activity patterns in the same experimental phase than between different phases. Gray areas show smoothed distribution densities of Euclidean distances. Boxplots show median, lower to upper quartiles with whiskers extending to the most extreme data points. Two-sided pairwise KS statistics applied. ***B***, Distributions of pairwise Euclidean distances of odor responses within the same animal and between different animals. More similar boutons activity pattern within the same animal than between different animals. ***C***, SNRs sorted according to odor type. Histogram of SNRs by odors. A Gaussian kernel density estimation of the underlying distribution was added. The orange line indicates the median of all SNRs that was used as a threshold to separate high and low SNRs. Download Figure 2-2, TIF file.

**Figure 3.**
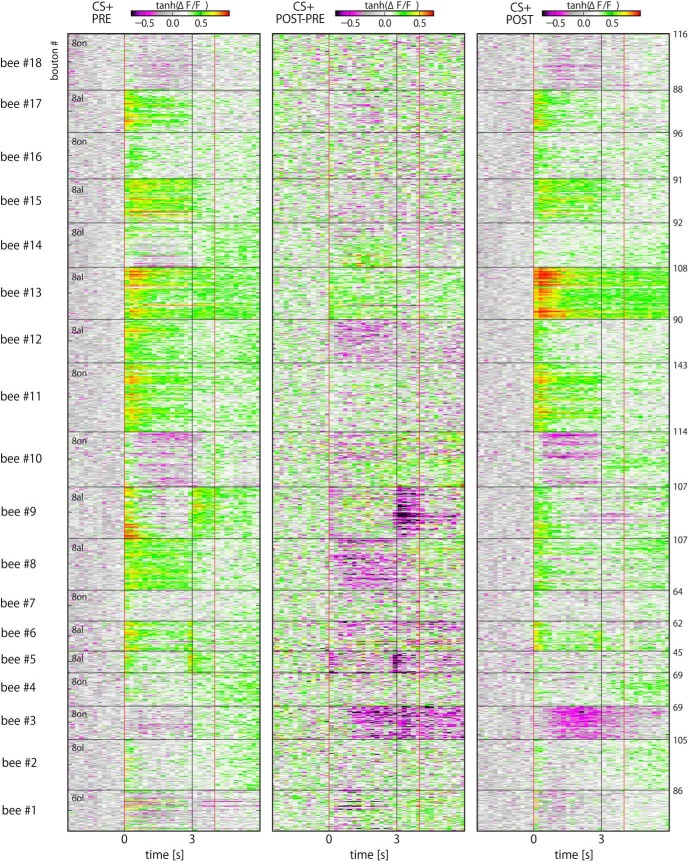
CS+ evoked responses in all recorded boutons. Color-coded CS+ odor responses of all 1652 PN boutons recorded from 18 bees. Ca^2+^ activity of individual boutons in PRE (left) and POST (right) phases as well as their differences (ΔNR, middle) are shown. Black horizontal lines separate boutons of different animals, whose IDs are given as ordinate labels. Odors were applied between 0 and 3 s. Measures of neural plasticity and SNRs were calculated on the basis of all time steps between 0 and 4 s (enclosed by red horizontal lines). 8ol: octanol, 6ol: hexanol.

10.1523/ENEURO.0128-18.2018.f2-3 Extended Data Figure 2-3 Decorrelation of spatial bouton response pattern. ***A***, Linear correlation coefficient R between the bouton response pattern to the CS+ odor and the CS- odor during the first second after stimulus onset PRE and POST training for all 18 bees. ***B***, The average correlation coefficient is smaller (*p* = 0.049, Wilcoxon signed rank test) after training (POST). ***C***, Average correlation coefficients before (PRE) and after (POST) training during spontaneous activity are close to zero and do not change from PRE to POST. Download Figure 2-3, TIF file.

Data were exported to Python or MATLAB (MathWorks Inc). Subsequent steps in the analyses were performed either using standard libraries for numerical/scientific programming in Python (NumPy, SciPy) of MATLAB. Overall, the observed Ca^2+^-dependent odor responses were dominated and overrepresented by excitatory signals. While an excitatory stimulation can lead to an arbitrary large increase in the Ca^2+^ signal, the inhibitory effect on the Ca^2+^ response is bounded, i.e., it can only suppress the relatively small amount of spontaneous Ca^2+^ activity. Therefore, before analyzing odor response profiles in more details, we transformed all data using the hyperbolic tangent function. The function provides a useful transformation to reduce the effect of outlying values of a variable while compressing large values (Extended Data Fig. 2-1; Godfrey, 2009). In our data, this transformation enhances small changes around zero in the Ca^2+^ signal and specifically improves the resolution of small inhibitory responses. This step facilitated the application of symmetric criteria for the classification of odor response profiles and their changes.

### Signal quality

To analyze pronounced odor-dependent variations in response strength and signal quality, the signal quality of each animal as its average signal-to-noise ratio (SNR) was calculated according to Equation 1, with $a_{b}$*(t)* representing the time-varying activity trace of bouton *b* for a total of $N_{b}$ boutons per animal. μ() and $\sigma^{2}$() indicate the mean and the variance, respectively:

(1) $$SNR_{b}=\left(\frac{P_{signal}}{P_{noise}}\right)=\frac{\mu \left(a_{b}{\left(t)\right|}_{t=0s}^{4s}\right)}{\sigma^{2}\left(a_{b}{\left(t)\right|}_{t=-2.6s}^{0s}\right)}SNR=\mu \left({SNR}_{b}{|}_{b=1}^{N_{b}}\right)$$

*SNRs* for each animal and stimulus type were calculated as an average of responses during both TRAIN and POST phases. Based on the bimodal distribution of these values we defined an empirical threshold to distinguish between weak (low *SNR*) and strong (high *SNR*) signals.

### ON and OFF responses

Excitatory and inhibitory ON and OFF responses were checked during respective response intervals, i.e., 1 s after odor on- and offset (five frames), for each bouton based on the trial-averaged Ca^2+^ activities (Table 1). During a baseline period of 2.6 s before odor onset, we computed the mean *a_0_* and SD $\sigma_{a0}$ across time. Excitatory responses were detected if the Ca^2+^ activity exceeded *a_0_* $\pm 2.5\sigma_{a0}$ in at least three of the five frames during the response interval. To capture the fewer, weaker and mostly delayed inhibitory ON responses, we extended the considered frames, requiring subthreshold values in at least three of the 15 frames whole during the 3-s odor presentation. A threshold of $2.5\sigma_{a0}$ proved to be suitable for separating the noisy signals that appeared during spontaneous activity from those values that are likely related to odor stimulation.

**Table 1.** Summary of odor response classes of PN boutons

	Types of excitatory odor responses				Types of response changes			
Stimulus	Only ON	Only OFF	ON and OFF	None	Only up	Only down	Both	None
CS+	39.5	3.3	9.9	47.2	14.2	33.0	7.8	45.0
CS-	40.9	2.7	12.3	43.6	18.7	31.4	5.7	44.2
Both	15.8	0.0	0.8	16.6	2.8	11.0	0.6	20.7

Types of excitatory odor responses of untrained animals: all 1652 boutons were categorized by their response profiles of (1) only ON responses, (2) only OFF responses, (3) both ON and OFF responses, or (4) no detected responses to CS+ and/or CS- odors. Types of excitatory bouton response changes by learning: all 1652 boutons were categorized by their response profiles of (1) only up-regulated, (2) only down-regulated, (3) both up- and down-regulated, or (4) no detected change to CS+ and/or CS- odors.

### Odor response plasticity

To categorize response plasticity into four classes (up, down, both, none; Table 1, right), positive and negative thresholds were set as *c_0_* $\pm 2.5\sigma_{c0}$, where *c_0_* and $\sigma_{c0}$ define the average and standard deviation of baseline activity of each bouton with respect to odor type (CS+, CS-). Bouton responses were then regarded as changed if the activity during and 1s after odor stimulation exceeded the threshold at least once during the time window. This threshold provided a suitable compromise for filtering most of the changes that appeared during spontaneous activity while keeping those that might be related to odor stimulation.

A single measure of neural response plasticity (ΔNR) was also assigned to each animal and stimulus type (Δ*NR* ^+^ for CS+ and Δ*NR* ^-^ for CS-). For this, we computed the sum of the absolute change across all frames that exceeded the aforementioned threshold on a per bouton basis. We then averaged the absolute change across boutons for each individual animal and stimulus type.

### Behavioral plasticity

The learning effect was quantified by calculating the change in the behavioral performances during the training phase (ΔCRTRAIN) and the POST training phase (ΔCRPOST) for each individual animal by computing the difference between its PER-activity in all but the first CS+ trials ${CR}_{t}^{CS+}$ and in the same number of CS- trials ${CR}_{t}^{CS-}$ divided by the number of trials $N_{t}$. Thus, ΔCR is the difference between the empirical probabilities of a CR *p(CR)* in CS+ and CS- trials.

$$\Delta CR=\frac{1}{N_{t}}\sum_{t=2}^{N_{t+1}}{CR}_{t}^{CS+}-\frac{1}{N_{t}}\sum_{t=1}^{N_{t}}{CR}_{t}^{CS-}(1.2)$$

For the training phase (TRAIN), we excluded the first CS+ trial from this calculation because only the subsequent trials of the training can be considered as measures in both acquisition and test trials. We defined bees as non-learners if they did not show a CR in any of the CS+ trials during both phases TRAIN and POST following the arguments presented by (Pamir et al., 2011).

### Correlation of behavioral and neural plasticity

We calculated Spearman's rank order correlation and the corresponding *p* value as a test for positive linear correlations between measures of behavioral performances (ΔCRTRAIN, ΔCRPOST) and neural plasticity (Δ*NR* ^+^, Δ*NR* ^-^). We performed correlation analyses separately for the full data set consisting of all 18 animals and for the subset of animals with recordings that showed high SNR according to the definition given in the previous paragraph (eight animals in the case of CS+ and 10 animals for CS-). Because of the low number of samples, we applied jackknife resampling as an estimate for the robustness and dependence on outliers of the correlation results.

### Bouton pattern correlation

For each bee, we computed the spatial bouton response pattern by averaging the Ca^2+^ activity within 1s after odor onset. The similarity of the response pattern between the CS+ and CS- odors before and after training was quantified by the linear correlation coefficient (*R*). We computed Fisher's z-transform of the *R* for the statistical comparison, and back-transformed to obtain the average linear correlation coefficient. We used a one-sided Wilcoxon signed-rank test under the null-hypothesis that the pattern correlation does not reduce by learning. For control, we compared CS+/CS- odor response pattern correlation during the baseline period of 2.6 s before odor onset.

## Results

### Behavioral performance of learning

We trained honeybees under the microscope in a differential conditioning paradigm as depicted in Figure 1*A*. One of two odors (CS+) was forward paired with the sucrose reward (US) while another (CS-) was presented without sucrose. We mainly used 2-octanone and octanal for odors (Fig. 1*B*). The performance of CR was evaluated by the probability of the proboscis extension response (PER) of the bee population to CS+ [p(CR); Fig. 1*C*]. Since the CS preceded the US by 2 s, memory acquisition during the training phase was also examined (TRAIN). None of the bees showed a PER in any of the odor stimulation trials before training (PRE). Bees that did not show a behavioral response to any of the CS+ trials in the training phase or the post training phase (POST) were classified as non-learners (five bees; see Materials and Methods). Including these non-learners, an asymptotic level of p(CR) ≈ 0.7 was reached after five rewarded trials. The response level decreased gradually during the 10 unrewarded extinction trials (five trials each for CS+ and CS-) in the POST phase (Fig. 1*C*, POST). We also observed PER to the CS- odor during training but not test, suggestive of a behavioral indication for CS- effect. A high behavioral performance during training phase implied a high performance after training phase (Pamir et al., 2014) with a significant correlation (ρ = 0.61, *p* < 0.007). We observed similar learning performances by different odors, indicative of rather good behavioral performances by bees even in this condition.

### Odor response profiles of PN boutons

To measure the odor-evoked Ca^2+^ responses from terminal boutons of a defined class of PNs in the MB calyx, the l-ALT PNs were dye-loaded with the Ca^2+^-sensitive dye fura-dextran via their somata (Fig. 2*A*). The dye was mixed with the lysine fixable dye tetramethylrhodamine-dextran that enabled to identify recorded neurons subsequently by a confocal microscopy (Fig. 2*B*,*C*). The activity patches were defined for the localizations of boutons in Ca^2+^ imaging data (Fig. 2*D*; see Materials and Methods; Yamagata et al., 2009).

Considering the noisy nature of the small activities of boutons, multiple trials of odor-evoked Ca^2+^ responses were examined before and after olfactory learning (Fig. 1*B*). Irrespective of the absolute signal strengths, nearly all of the recorded animals showed odor- and phase-specific spatial response patterns across boutons (Fig. 2*E*), a finding expected from the combinatorial odor processing and associative plasticity in the AL and the MB (Joerges et al., 1997; Faber et al., 1999; Faber and Menzel, 2001; Szyszka et al., 2005; 2008; Krofczik et al., 2008; Fernandez et al., 2009; Yamagata et al., 2009; Galizia and Rössler, 2010; Rath et al., 2011; Arenas et al., 2012). The trial variances of these responses were reasonably small such that the Euclidean distances of Ca^2+^ responses between given two trials within the same phase (i.e., PRE or POST) were significantly smaller than that between phases [i.e., PRE vs POST; Extended Data Fig. 2-2*A*; Kolmogorov–Smirnov (KS) test, *p* < 0.0001]. Accordingly, we conducted further analyses based on the trial-averaged bouton activities to improve the S/N.

To acquire an overview of the odor response profiles of boutons in the untrained condition, we categorized their responses (ON, OFF, ON and OFF, and none) by stimulus type (CS+, CS-, and both; Table 1; see Materials and Methods). Although boutons were visually identified by the responses (Fig. 2*D*), ∼17% of the activities appeared too sparse or too noisy to satisfy our rather conservative criteria (none and both group). The fraction of ON, OFF, and ON and OFF responses were roughly equally distributed in both odors to be CS+ and CS-, suggestive of their similarity in odor responses before learning. 1-Octanal elicited generally stronger odor responses than 2-octanone, which is represented as the bimodal distribution of signal qualities (Extended Data Fig. 2-2*C*).

**Figure 4.**
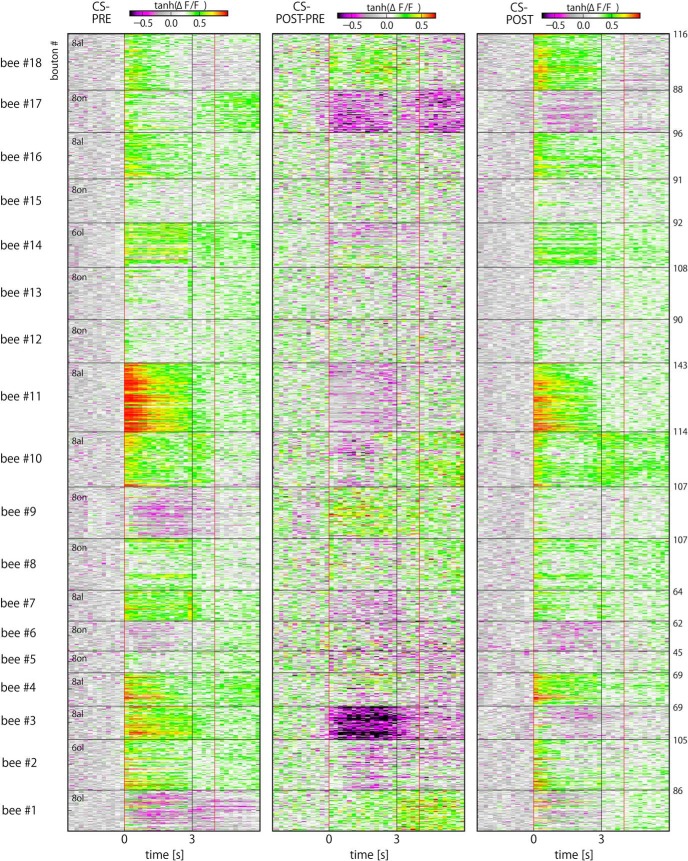
CS- evoked responses in all recorded boutons. Color-coded CS- odor responses of all 1652 PN boutons recorded from 18 bees. Same as in Figure 3.

**Figure 5.**
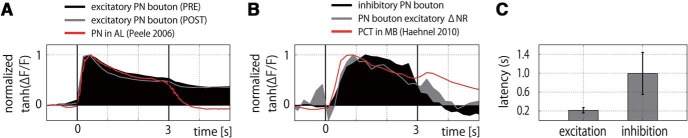
Temporal profiles of odor-evoked PN bouton activity. ***A***, Time courses of the mean excitatory responses (CS+ and CS- pooled) of PRE (black) and POST (gray) phases with previously published Ca^2+^ imaging data of PNs (red; Peele et al., 2006; *n* = 112). ***B***, Time courses of the mean inhibitory response (black) and the difference in excitatory responses (ΔNR/PRE-POST, gray) with previously published Ca^2+^ imaging data of MB feedback neurons (red; Haehnel and Menzel, 2010; *n* = 30). ***C***, Mean latencies of excitatory and inhibitory responses relative to valve opening of the odor stimulation device.

Temporal properties of odor responses were analyzed by calculating time-resolved activities for each bouton. Individual bouton response traces of a representative bee are exemplified in Figure 2*F*. A measure for neural plasticity (Δ*NR*) was calculated by taking the differences between PRE and POST phases. Figures 3, 4 show all recordings of 1652 boutons from 18 bees in response to CS+ (Fig. 3) and CS- (Fig. 4) odors. The boutons typically exhibited phasic-tonic activities that may or may not last longer than an odor presentation. Bouton response patterns were rather homogeneous within an animal but diverse across animals (Figs. 3, 4). Accounting for this, the Euclidean distances of individual bouton activities within an animal were significantly smaller than those between animals (KS test, *p* < 10^−4^; Extended Data Fig. 2-2*B*). Within the first second after odor onset, PN bouton activities were clearly dominated by excitatory responses (Figs. 3, 4). In contrast, inhibitory responses of boutons arose with rather slower kinetics. We compared their latencies, i.e., the first time point of the Ca^2+^ traces that exceeded the defined thresholds (see Materials and Methods). Most excitatory responses started within the first two frames after odor onset with an average of 0.22 s (Figs. 5*A*,*C*) while that of inhibitory responses distributed more widely and were much slower, with an average of ≈1 s (Figs. 5*B*,*C*). These distinction suggest separated mechanisms for excitatory and inhibitory inputs onto the PN boutons, presumably through feed-forward excitatory inputs mainly from PN axons and feed-back inhibitory inputs mainly from GABAergic PCT neurons (Grünewald, 1999a; Filla and Menzel, 2015).

### Plasticity of bouton activities and their correlation with behavior

We sought for neural correlates of learning behavior in the PN boutons. To grasp a coarse overview of the neural plasticity that takes place in the boutons, we first categorized boutons by their response profiles during learning (Table 1). A majority (∼50%) of them changed the responses only to one direction, i.e., either increased or decreased their responses, while ∼13% of boutons showed changes in both directions. Response reduction appeared more often (>30%) than response increase, suggesting a dominant learning effect through some inhibitory mechanisms. The probability of each type of change was roughly equal between CS+ and CS- odors, suggesting no apparent contrasts between rewarded and unrewarded odors in this regard. We therefore tested whether the overlap between the spatial bouton response patterns to the CS+ and CS- odors is reduced as a result of learning (see Materials and Methods) and found that, indeed, there is a significant reduction (*p* < 0.05, Wilcoxon signed rank test) of the response pattern correlations with an average reduction from *R* = 0.30 to *R* = 0.22 (Extended Data Fig. 2-3).

**Figure 6.**
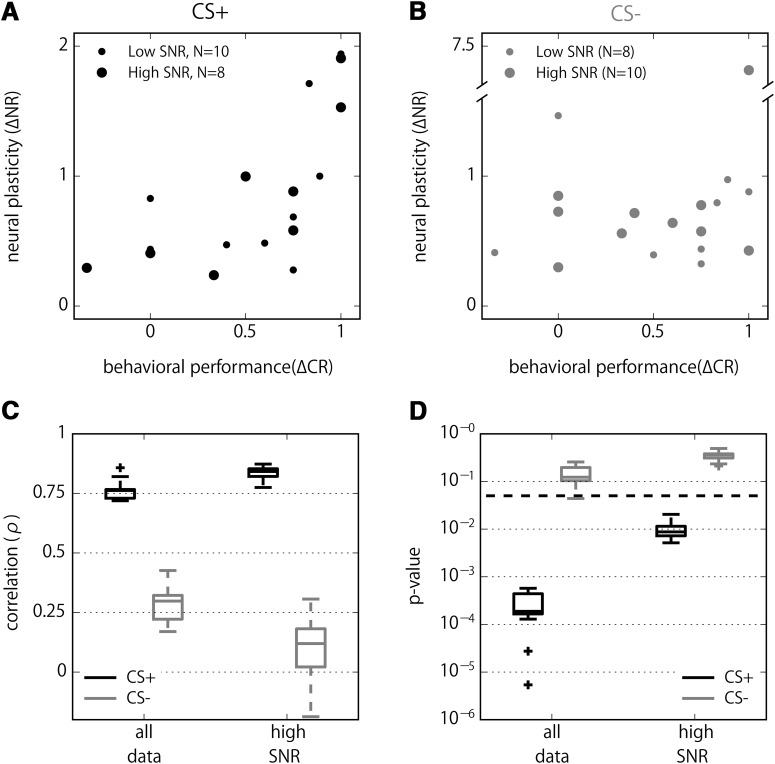
Positive correlation between a conditioned behavior and bouton plasticity. ***A***, ***B***, Scatter plots of behavioral performance during training phase (ΔCR) against neural plasticity (ΔNR) for CS+ (***A***) and CS- (***B***) odors. ***C***, Jackknife resampling of Spearman's rank order correlations (ρ) on high SNR (*n* = 8 for CS+, *n* = 10 for CS-) and full data (*n* = 18) to estimate robustness and dependence on outliers. ***D***, Corresponding *p* values of ***C***. Thick dashed line indicates a level of significance of 0.05.

We hypothesized that the neural plasticity in the PN boutons correlates with the behavioral plasticity. We assigned the unsigned neural plasticity Δ*NR,* which only considers absolute changes of bouton activities (see Materials and Methods). Likewise, we quantified the changes in behavior (Δ*CR*) of each individual bee during and after training (see Materials and Methods). We found a clear and highly significant positive correlation between learning induced neural plasticity of CS+ odor and behavioral performance (Fig. 6*A*; ρ = 0.76, *p* < 0.0002). This correlation was even more pronounced for a subset of animals that showed Ca^2+^ responses with a high SNR (see Materials and Methods; ρ = 0.84, *p* < 0.004). In contrast, the CS- plasticity did not show a significant correlation with the behavior (Fig. 6*B*), neither when considering all animals (ρ = 0.29, *p* > 0.12) nor for a subgroup of high SNR bees (ρ = 0.09, *p* > 0.39).

The robustness of the correlation was confirmed by a jackknife approach, where neural and behavioral data from all but one animal was iteratively resampled (Fig. 6*C*,*D*). Irrespective of signal SNR, the correlation of CS+ odor was narrowly distributed around the above stated values. The corresponding *p* values suggested statistical significance in all cases. In contrast, CS- odor exhibited much lower correlations with high *p* values. Taken together, we concluded that the observed plasticity in the PN bouton correlates with the learned value of a stimulus to alter the behavior of the bees.

Finally, we also assessed the plasticity in temporal dynamic of odor responses by calculating the difference of excitatory odor response between the PRE and POST phases (Fig. 5*B*). Interestingly, the time course resembled temporal kinetics of the odor response of the MB extrinsic feedback neurons previously reported (Haehnel and Menzel, 2010). This finding suggests that the observed plasticity in the PN boutons can be explained by the altered input strength from the inhibitory feedback neurons onto the boutons.

## Discussion

Here, we studied neural plasticity of the PN boutons in the MB calyx in relation to the learning rate at the level of the individual bee. To this end, we combined Ca^2+^ imaging and associative olfactory learning. A majority of bees readily performed learning behavior under our experimental conditions (Fig. 1). Both at PRE and POST phases, odor-unique and repetitive spatiotemporal population responses of PN boutons were observed (Figs. 2–4) as shown previously (Szyszka et al., 2005; Yamagata et al., 2009). We found upregulation and downregulation of bouton activities to CS+ and CS- odors, which occurred equally frequently by odors (Table 1). The absolute amount of neural plasticity was strongly and positively correlated with the behavioral learning success across individuals for the CS+ but not the CS- odor (Fig. 6). The absence of correlated neural plasticity in CS- may be relevant to (1) the neural plasticity linked to the behavioral CS- effect (Fig. 1*C*); (2) the hidden neural adaptations that are not directly expressed in behavioral change, e.g., retardation of reversal learning or decreased generalization in the course of differential conditioning (Extended Data Fig. 2-3; Menzel et al., 2007); and (3) neural changes that are not accompanied with behavioral changes, which is exemplified and well documented with neural activity in PCT neurons in the MB during PER conditioning to visual cues or visual context (Filla and Menzel, 2015). Moreover, the temporal profile of the induced changes by odors matched that of the inhibitory GABAergic PCT neurons (Haehnel and Menzel, 2010), suggestive of a functional linkage between PN boutons and PCT neurons for learning. We thus hypothesize that the observed plasticity in olfactory presynaptic terminals of the PN boutons relates to the learned value of the stimulus, signifies short-term memory and underlies the CR behavior.

Each single bouton of PN receives olfactory input from the AL and forms the core of a microglomerular structure by contacting with several postsynaptic KCs, inhibitory presynaptic feedback PCT neurons and modulatory VUM reward neurons (Hammer, 1993; Ganeshina and Menzel, 2001; Schröter and Menzel, 2003; Sinakevitch et al., 2013). These neuroanatomical properties suggest a separate regulation of excitatory and inhibitory inputs onto the PN boutons. Indeed, the majority of the excitatory responses match the phasic-tonic profiles and low response latencies of PNs, known from previous Ca^2+^ imaging data recorded in PNs of the AL (Peele et al., 2006; Fig. 5*A*) and the typical intracellularly and extracellularly recorded spike responses in PNs (Krofczik et al., 2008; Strube-Bloss et al., 2011; Brill et al., 2013). The average inhibitory response, on the other hand, matches previously recorded data from inhibitory PCT neurons (Haehnel and Menzel, 2010; Fig. 5*B*). Interestingly, the difference between the average excitatory profiles (PRE-POST) is closely related to the temporal structure of the average inhibitory response and also resembles those observed in the PCT neurons (Haehnel and Menzel, 2010; Fig. 5*B*). Thus, decreased responses in the PN boutons are likely to be the consequence of activity modulation by the GABAergic PCT neurons. Likewise, a part of activity increase in boutons may also be explained by the inhibitory modulation of odor responses of PCT neurons on learning (Grünewald, 1999b). In bees, GABAergic signaling in the MB calyces are reported to be important for the discrimination of similar odors (Devaud et al., 2015). It is thus conceivable that our observed plasticity in the PN boutons contributes to enhance odor discriminability (Extended Data Fig. 2-3), thereby underpinning an odor-specific CR.

The clear correlation between the absolute amount of plasticity with the behavioral change (Fig. 6) highlights the importance of both upregulation and downregulation of bouton activities in learning. Seen in the context of the olfactory pathway in insects, olfactory memory is distributed across various sites: from the pre- and postsynaptic sites in the glomeruli of the AL to the pre- and postsynaptic sites in the MB input (the lip of the calyx), and the MB output lobes (Heisenberg, 2003; Menzel, 2012). In those neural circuits in bees, the increase and decrease of neural changes for the learned stimulus is a wide spread property of neurons. For example, spike activity recorded extracellularly from PNs increase or decrease their responses to the learned odor with similar probability (Denker et al., 2010), which potentially obscures associative changes as recorded with Ca^2+^ imaging at the postsynaptic sites in AL glomeruli (Peele et al., 2006). Fernandez et al. (2009) and Rath et al. (2011) also found that the total response strength of PNs measured as Ca^2+^ signals does not change after conditioning although the response patterns changed. Although MB KCs predominantly increase their responses for the CS+ and decrease it for the CS-, also respective opponent changes were found (Szyszka et al., 2008). MBONs are well known for their opponent changes in the course of learning for both an olfactory cue and a visual context (Haehnel and Menzel, 2010; Strube-Bloss et al., 2011, 2016; Filla and Menzel, 2015). Similar opponent changes were also found in MBONs in *Drosophila* (Cohn et al., 2015; Owald et al., 2015). Neural plasticity without altering overall excitation may therefore be a general mechanism seen in the insect brain, presumably to achieve effective memory encoding in the limited coding space with less energy consumption.

Although we could not image the boutons during the acquisition phase due to technical limitations, we assume that synaptic plasticity develops already during acquisition. This idea is supported by the finding of neural changes already 15 min after the last training trial. Hammer and Menzel (1998) also found that US substitution by octopamine injection into the MB calyx paired with an odor leads to the acquisition of appetitive memory. The synaptic plasticity observed in PN boutons is likely to be a component of short-term memory (Menzel, 1999, 2012). In this respect, the olfactory input to the MB shows characteristics of some particular MB extrinsic neurons, e.g., the PE1 neuron (Mauelshagen, 1993; Okada et al., 2007) and other A1/A2 output neurons (Menzel and Manz, 2005) but not of all MB extrinsic neurons that may develop their learning-dependent plasticity only 3 h after the last learning trial (Strube-Bloss et al., 2011) or even later (Haehnel and Menzel, 2012). Both short-term memory and consolidation to long-term memory may thus be the characteristics of the MB, although their neuroanatomical substrates may not overlap completely. In flies, a segregation of local circuits within the MB is well established for short-term and long-term memory storage and encoding (Trannoy et al., 2011; Yamagata et al., 2015, 2016).

Within each of the animals, the temporal structures of individual bouton responses were often closely related to each other. Due to technical limitations, we observed a specific, locally restricted patch of the MB calyx of each animal, comprising an estimated 8% of the total area. We may thus assume that this small fraction of calyx consists of boutons from a few and possibly similarly tuned PNs. Recent evidence from the fruit fly suggests that PNs with similar tuning properties have a tendency to converge onto the same KCs (Gruntman and Turner, 2013). PNs also form multiple boutons along their axons in the calyces (Zwaka et al., 2016), which exhibit correlated activity to odors (Yamagata et al., 2009).

While we observed animals with a low neural plasticity score Δ*NR* ^+^ that also showed a stable behavioral performance Δ*CR*, we did not observe animals with a high neural plasticity score accompanied by a weak behavioral performance (Fig. 6*A*). A likely explanation is that we captured only a fraction of the neural changes that appeared in a specific animal due to the undersampling of a small fraction of the calyx. In addition, the staining procedure may have resulted in a varying amount of backfilled PNs. Presumably through these and other related issues, the quality of odor-evoked signals differed across preparations and for different odors, and physiologic changes may have been concealed. These reasons can lead to a reduced but not to an overestimated Δ*NR* ^+^, which could explain the observation in Figure 6*A*.

In the MB calyx, associative plasticity is conveyed by multiple network properties, i.e., modulation of the presynaptic sites in the AL glomeruli of PNs (Faber et al., 1999; Fernandez et al., 2009; Rath et al., 2011), the postsynaptic sites in the spines of the MB intrinsic KCs (Szyszka et al., 2008), and the postsynaptic sites of the inhibitory feedback neurons in the PCT (Grünewald, 1999b; Haehnel et al., 2012; Filla and Menzel, 2015). The octopamine immunoreactive reward pathway of the VUM neuron with presynaptic terminals to both the PN boutons and the KC spines (Ganeshina and Menzel, 2001; Sinakevitch et al., 2013) also changes its response properties to the forward paired odor (Hammer, 1993). We emphasize that the observed plasticity in the PN boutons is not a simple copy of these neural correlates of olfactory memory by the following reasons. (1) Local synaptic profiles surrounding the PN boutons receive octopaminergic modulatory inputs from the VUM neurons (Ganeshina and Menzel, 2001; Sinakevitch et al., 2013), indicative of their capability as a site of inducing associative plasticity. Indeed, Hammer and Menzel (1998) demonstrated that a focal microinjection of octopamine into the MB calyx paired with an odor leads to the formation of appetitive memory. In *Drosophila*, microglomeruli in the MB calyx constructed by PN boutons, KC spines and GABAergic interneurons (Yasuyama et al., 2002; Butcher et al., 2012) also undergo activity-dependent physiologic plasticity (Pech et al., 2015). (2) Boutons of the PNs are the convergence site of two forms of learning related plasticity (i.e., from the AL via PNs and from the MB Lobes via PCTs), certainly storing different contents from the memory in these brain regions. Thus, the PN boutons are expected to act as a hub of multiple memory information and sensory inputs. It is, therefore, reasonable to see associative plasticity in the PN boutons in both increasing and decreasing manner, presumably depending on which of its inputs (excitatory from the AL, inhibitory from recurrent neurons) are more effective. Although a clear correlation between conditioned behavior and neural plasticity (Fig. 6) suggests a specific role of PN boutons for mediating learned value of odors, it may not be the sole function of the microglomeruli. In bees, experience and age-dependent structural plasticity has been reported (Groh et al., 2012; Muenz et al., 2015), indicating a convergence of state information and a resultant circuit rewiring. Future studies employing a state-of-the-art genetic technique that makes it possible to target specific synapses (Hayashi-Takagi et al., 2015) would provide valuable insights into the functional significance of this unique microcircuitry.

## Synthesis

Reviewing Editor: Tatyana Sharpee, The Salk Institute for Biological Studies Decisions are customarily a result of the Reviewing Editor and the peer reviewers coming together and discussing their recommendations until a consensus is reached. When revisions are invited, a fact-based synthesis statement explaining their decision and outlining what is needed to prepare a revision will be listed below. The following reviewer(s) agreed to reveal their identity: André Fiala, Paul Szyszka.

This study reports important new results on functional synaptic plasticity in the presynaptic sites of the mushroom body calyx induced by classical odor-sugar conditioning. The authors find a correlation between the amount of neural plasticity and behavioral learning performance. Relatively minor comments need to be addressed as described below.

Reviewer 1:

- The authors show that the degree of neuronal plasticity correlates with the degree of behavioral plasticity, but only for the CS+. This is not surprising, because the animals do not change their behavior to the CS-. However, the degree of neuronal plasticity for the trained CS- seems to be the same as for the CS+. This should be critically discussed, and the data for neuronal plasticity induced by CS+ and CS- should be compared! Apparently, learned behavior and neuronal plasticity do not always correlate, and one would not expect that.

- Correlations are never indicative for causal connections, and this is also the case here. The data do not show that the neuronal plasticity observed here is causative for the learned behavior. This must be made very clear, and statements like, e.g., “the observed plasticity...underlies the conditioned response behavior” (page 12, line 35) must be removed.

- In the Materials and Methods section, chapter “Odor stimulation and conditioning” it should be stated early on that it is the proboscis extension reflex that is observed. I was wondering what ‘sugar response’ (page 4, line 111) actually means until I found the explanation on page 5, line 121.

- Please explain all abbreviations in the figures also in the legends, e.g., figure 2 ‘8on’ and ‘8al’. Why not writing boutons instead of ‘selected locations’? Is the bee shown in figure 2 bee # 11 in figure 3? Please indicate that this is one example of one representative bee.

- Please also explain in more detail what tanh(ΔF/F) means, and how the data after applying this function relate to the original fluorescence changes. This is of importance! Perhaps showing the original florescence change values is helpful to understand this calculation step.

- Abstract: the authors do not show memory traces, as suggested by the first two lines, but correlations. Line 7: the conditioned response is in this case the conditioned behavioral response! Please avoid terms like ‘clearly correlated’ (line 8): either there is a correlation or not. The terms ‘activity plasticity’ and ‘activity reshape’ are not clear.

- Significance statement: The first two sentences on insects in general are too speculative. Also, the amount of plasticity correlates with the learned behavor, but only for the CS+, not for the CS-! Also, the verb ‘unprecedented’ evidence is too strong. There is evidence that PNs undergo plasticity, and the calcium changes observed in the calyx might reflect plasticity in the antennal lobe. This should be discussed as well.

- Introduction: the first paragraph explains that several neuropils (AL and MB) show learning-related plasticity. Please make clear that these statements do not refer to insects in general or all kinds of learning. Rather, evidence exists for the honeybee and sugar-reward-based associative conditioning of the PER. The citation Thum et al. 2007 on Drosophila is not correct in this context (shows a requirement of rutabaga in PNs for reward learning only).

- Please be accurate in explaining the role of the VUMmx1 neuron. This neuron is not the sugar pathway (e.g., page 2, line 50, or page 2, line 48). It is one neuron that is sufficient to signal the reiforcing properties of the rewarding unconditined stimulus.

- Please do not mix up Drosophila and honeybees. I suggest deleting all Drosophila citations from the introduction and thoroughly and accurately discussing the differences between both model organisms and the learning paradigms in the discussion section. The typical learning paradigms the authors probably refer to are just very different, and one cannot assign learning and memory in general to a specific brain location in either organism. In fact, the idea that a PCT neuron backpropagating from the lobes to the calyx transmit learned information would place the site of initial plasticity to the lobes, and not to the calyx, in agreement with current models of reward learning in Drosophila!

Reviewer 2:

This is a well done study which adds important new information about the mechanisms of odor learning, the paper is well written and exciting.

I have only minor suggestions (feel free to adopt them):

There are several papers that show that the similarity between a CS+ and CS- induced activity patterns decreases after odor-reward conditioning in the AL and in PNs (Faber et al., 1999; Fernandez et al., 2009; Rath et al., 2011). I would test, whether this is also the case in the PN boutons.

I would discuss the odor-reward conditioning-induced decrease in odor-evoked PCT responses (Gruenewald 1999) - how does this relates to your findings?

Line 3-5: I would be more specific about the kind of learning and change “The mushroom body (MB) in insects is known as a major center for learning and memory” to “The mushroom body (MB) in insects is known as a major center for associative learning and memory” and “„undergo activity plasticity through learning and correlate with the conditioned behavior” to “undergo activity plasticity through classical odor-reward conditioning and correlate with the conditioned behavior”

Line 6: I would change “We simultaneously measured Ca2+ responses in the boutons and conditioned responses of the bees to learned odors” to “We simultaneously measured Ca2+ responses in the boutons and conditioned responses to learned odors in honeybees” (because ‘the bees’ were not mentioned before)

Line 8: I would remove clearly from “specifically for the rewarded odor was clearly and positively correlated with the behavioral learning success across individuals” because ‘clear’ is ill-defined.

If “specifically for the rewarded” means “for the rewarded but not for the unrewarded odor” then I would write “for the rewarded but not for the unrewarded odor”.

Lines 21, 24: I would remove ‘substantial’ because it is clear that your work is substantial. Stating that it is substantial is unnecessary and it might provoke the question ‘what exactly is substantial’.

Line 41: I would change “olfactory relay projection neurons (PNs)” to “olfactory projection neurons (PNs)” or “olfactory relay (projection) neurons (PNs)”

Line 48: I would change “octopaminergic reward pathway that conveys the rewarding sugar information during learning (Hammer, 1993).” To “octopaminergic neuron that conveys the rewarding sugar information during associative learning (Hammer, 1993).”

Line 53: I would remove “have been repeatedly demonstrated to”

Line 55: Strube et al., 2011 (10.1523/JNEUROSCI.2583-10.2011) should be added to: “Mauelshagen, 1993; Grünewald, 1999b; Okada et al., 2007; Filla and Menzel, 2015”

Line 59: ‘learning specific’ should be ‘learning-specific’

Line 90: Did you inject a liquid or solid dye?

Line 105: How did you dilute the odorants?

Line 108: Was this the same odor delivery device used by Peele 2006 and Hähnel 2010? If yes I would mention it here (or later), because the time-course of Ca2+ responses reflects the stimulus time course and you compare your Ca2+ time courses with that of Peele and Hähnel.

Line 109: What is the difference between “inter-stimulus interval and an inter-trial interval”?

Line 121: What was the criterion for a PER?

Line 127: You alternate between ‘calcium’ and ‘Ca2+’. I would only use ‘Ca2+’.

Line 168: I would illustrate (either in words or with a suppl. Figure) what exactly happens here: “we

transformed all data using the hyperbolic tangent function”. E.g., show some Ca2+ signals before and after this transformation.

Line 207: What does N and R in ‘ᵄ1;ᵄ5;’ stands for?

Line 243: I would change “was unpaired with sucrose” “was presented without sucrose”

Line 270: There are two earlier studies that found odor-specific spatial response patterns across PN boutons in the lip which should be added to the reference: Szyszka et al., 2005 (10.1152/jn.00397.2005) and Yamagata et al., 2009. And Faber et al. 2001 is missing who showed learning-induced plasticity in the MB.

Line 284: I think it should be 2-octanone (with e)

Line 348: “spatio-temporal population responses of PN boutons were observed (Figs. 2-4) as shown previously (Yamagata et al., 2009)”: I would add (Szyszka et al., 2005) who also found spatio-temporal population responses of PN boutons.

Line 386: “For example, spike activity recorded extracellularly from PNs increase or decrease their responses to the learned odor with similar probability (Denker et al., 2010)”: I would add the information that also Fernandez et al., 2009 (Fig. S4) and Rath et al., 2011 (Fig. 2A) found that the total response strength of PNs (measured as Ca2+ signals) does not change after conditioning - even though the response patterns change.

Figure 2e: Are the latencies relative to valve-opening or to odorant arrival at the antennae?

Figure 3 and 4: I would sort the bees according to their learning performance.
