# Supplementary figures and images for: Neural Correlates of Odor Learning in the Presynaptic Microglomerular Circuitry in the Honeybee Mushroom Body Calyx
Source: eNeuro. 2018 Jun 18;5(3):ENEURO.0128-18.2018. doi: 10.1523/ENEURO.0128-18.2018 (PMC6011417; doi:10.1523/ENEURO.0128-18.2018)

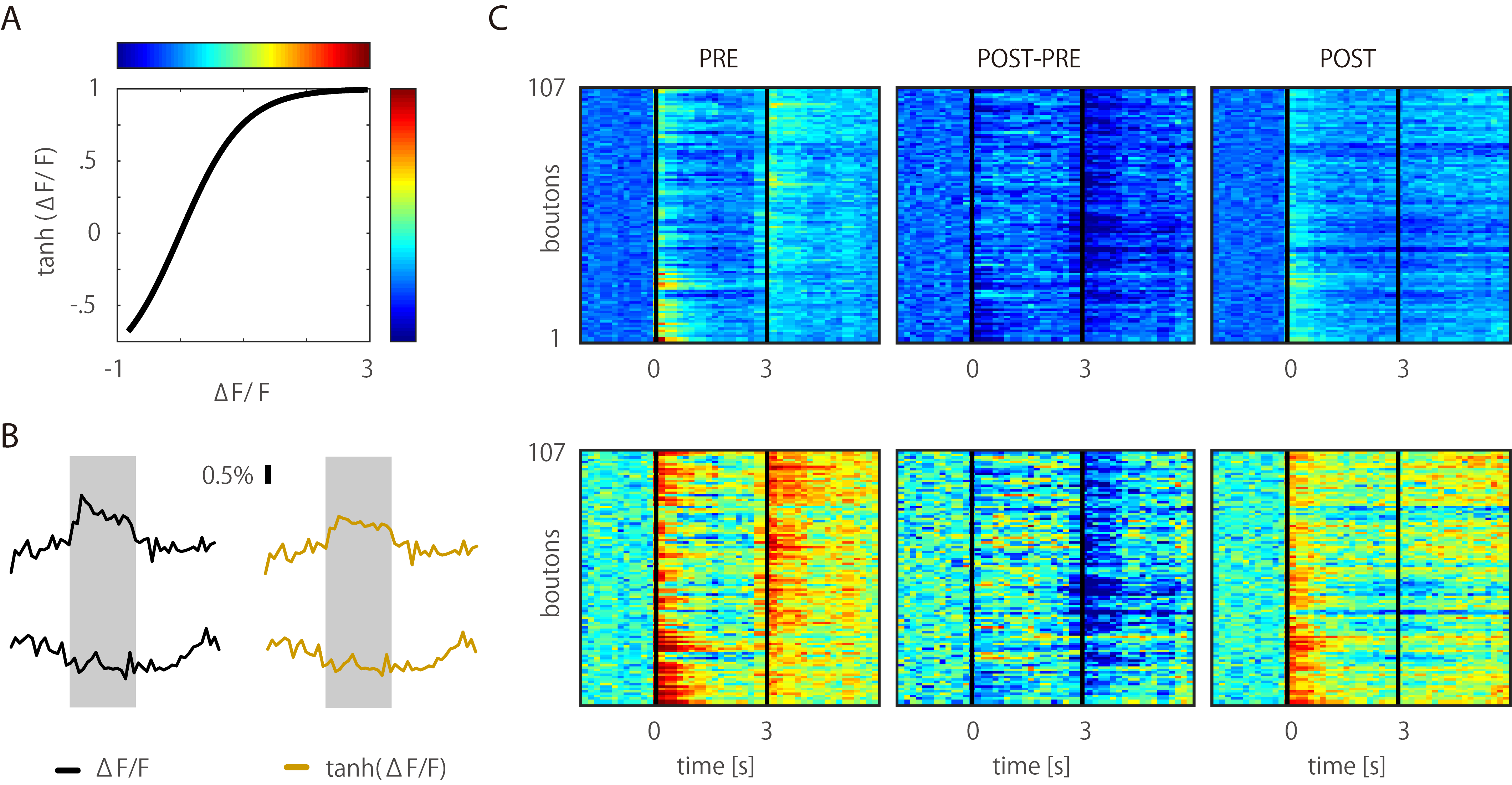

Supplement: Extended Data Figure 2-1 — Transformation of Ca2+ responses. A, Tangens hyperbolicus transformation of ΔF/F signal. Black curve represents transformation across the total range of ΔF/F values encountered in all boutons of bee #9. B, Transformation of exemplified single bouton activities. Black shades indicate odor stimulation for 3 s. Large excitatory response values are compressed by the transformation while smaller excitatory and inhibitory response values around zero are enhanced. C, Color code visualization of all 107-bouton response in bee #9 in response to the CS+ odor before (PRE) and after (POST) training and the difference (POST-PRE). The top row shows the untransformed ΔF/F response signals. The color map spans the complete data range across all boutons as shown in A, top. The bottom row shows the transformed tanh (ΔF/F) response signals. The color map spans the complete range of transformed data across all boutons as shown in A, right. This leads to a compression of strong excitatory responses (reddish colors) and increases the dynamic color range for weaker excitatory and inhibitory responses. In effect, we observe clear excitatory and inhibitory responses across many boutons. The same classical “jet” 64-bit color map (as shown in A) is used for upper and lower row. Download Figure 2-1, TIF file. [file sup_enu-eN-NWR-0128-18-s01.tif]

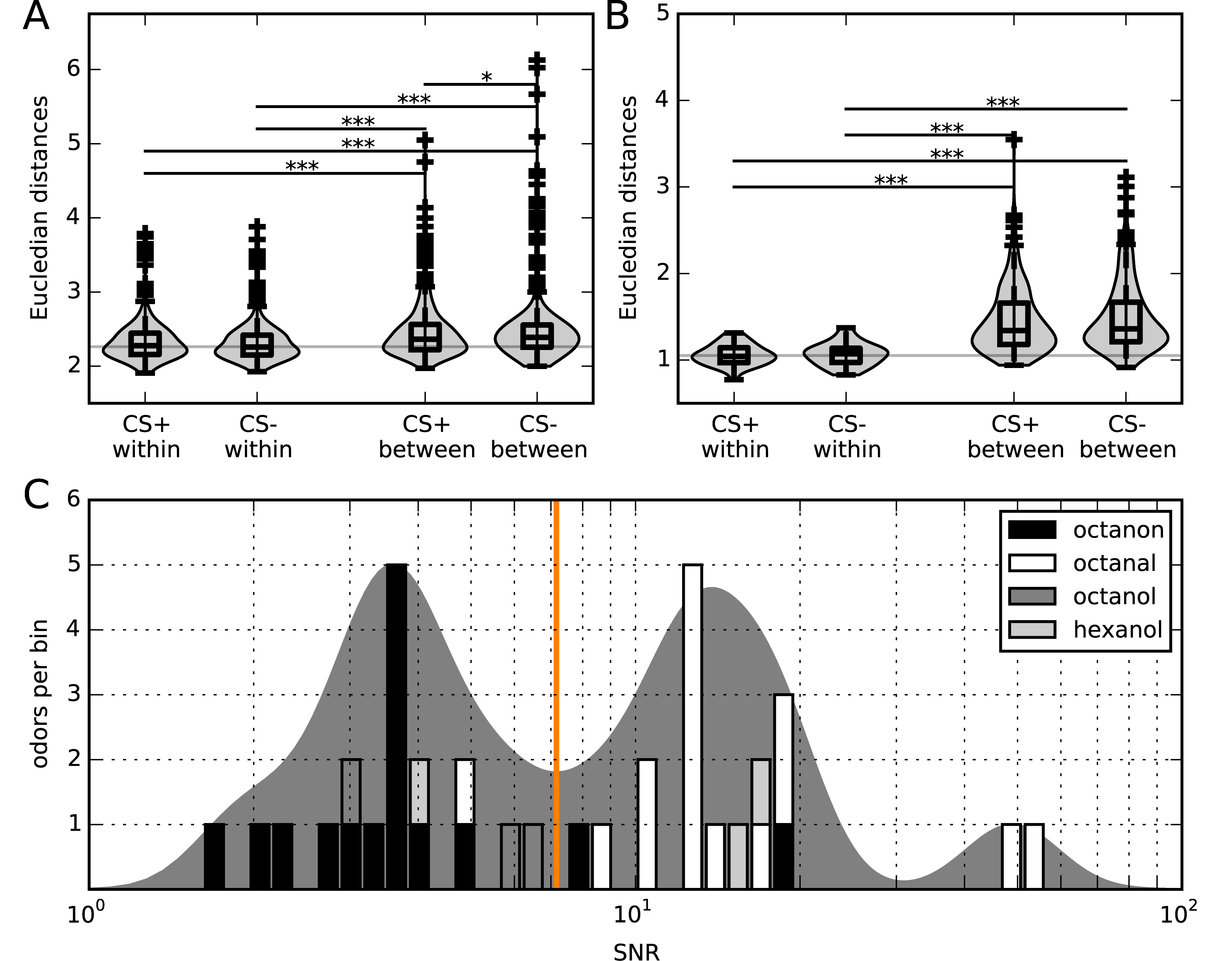

Supplement: Extended Data Figure 2-2 — Response statistics within and across experimental phases. A, Distributions of pairwise Euclidean distances of odor responses of different trials within and between different experimental phases. More similar bouton activity patterns in the same experimental phase than between different phases. Gray areas show smoothed distribution densities of Euclidean distances. Boxplots show median, lower to upper quartiles with whiskers extending to the most extreme data points. Two-sided pairwise KS statistics applied. B, Distributions of pairwise Euclidean distances of odor responses within the same animal and between different animals. More similar boutons activity pattern within the same animal than between different animals. C, SNRs sorted according to odor type. Histogram of SNRs by odors. A Gaussian kernel density estimation of the underlying distribution was added. The orange line indicates the median of all SNRs that was used as a threshold to separate high and low SNRs. Download Figure 2-2, TIF file. [file sup_enu-eN-NWR-0128-18-s02.tif]

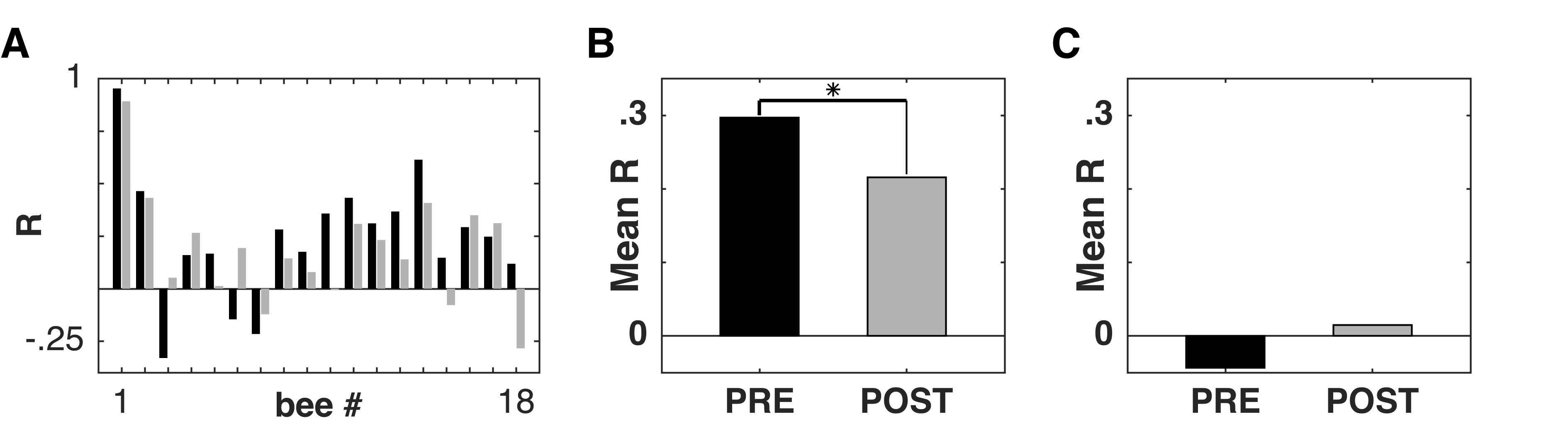

Supplement: Extended Data Figure 2-3 — Decorrelation of spatial bouton response pattern. A, Linear correlation coefficient R between the bouton response pattern to the CS+ odor and the CS- odor during the first second after stimulus onset PRE and POST training for all 18 bees. B, The average correlation coefficient is smaller (p = 0.049, Wilcoxon signed rank test) after training (POST). C, Average correlation coefficients before (PRE) and after (POST) training during spontaneous activity are close to zero and do not change from PRE to POST. Download Figure 2-3, TIF file. [file sup_enu-eN-NWR-0128-18-s03.tif]
